# Vegetable intake and major depressive disorder: insights and future directions for nutritional psychiatry

**DOI:** 10.1017/S1368980024002714

**Published:** 2025-01-07

**Authors:** John Patrick C Toledo

**Affiliations:** Department of Theology and Religious Education, De La Salle University, 2401 Taft Avenue, Manila 1004, Philippines

**Keywords:** National Health, Vegetable, Mendelian randomisation, Major depressive disorder

Dear Editor,

The study titled^(1)^ ‘Association between vegetable intake and major depressive disorder: results from National Health and Nutrition Examination Survey 2005–2018 and bidirectional two-sample Mendelian randomisation’ uses cross-sectional and Mendelian randomisation techniques to give a thorough examination of the relationship between vegetable consumption and major depressive disorder (MDD).

The authors found a significant relationship between low vegetable intake (defined as less than two cups per d) and an increased risk of MDD in a cross-sectional analysis involving 30 861 US adults. According to the study, the OR for developing MDD associated with low vegetable intake was 1·53, with a 95 % CI of 1·32, 1·77, indicating a compelling association. A causal association was not supported by the bidirectional two-sample Mendelian randomisation analysis, indicating that although there is a substantial correlation, the directionality of the relationship is still unclear.

The study’s utilisation of thorough NHANES data and sound statistical methodology are among its many important strengths. Notwithstanding corrections for different demographic characteristics, the cross-sectional design has disadvantages because it precludes causal inference and possible residual confounding. Furthermore, more research is required to determine the precise consumption amounts influencing mental health outcomes due to the non-linear association that was found.

Future research in this area should use longitudinal designs to further determine causation and examine the various effects of eating raw *v*. cooked vegetables on mental health outcomes. A systematic review highlighted that dietary patterns, particularly the intake of fruits and vegetables, are associated with mental health outcomes over time^(2)^. Jack, Cherbuin, Anstey and Butterworth in their article entitled *Does reverse causality explain the relationship between diet and depression? Journal of Affective Disorders* in the *Journal of Affective Disorders,* which emphasised the importance of longitudinal data to establish clearer causal relationships between diet and mental health, suggesting that future studies should consider the preparation methods of food, including raw *v*. cooked forms. Furthermore, increasing the demographic diversity of research subjects may improve the findings’ generalisability. In order to inform dietary suggestions targeted at decreasing the risk of MDD, future research must also explore the individual elements found in vegetables that may moderate these connections. Overall, this study emphasises the significance of vegetable diets for mental health and makes a significant contribution to our understanding of nutritional psychiatry. This strategy may result in dietary recommendations that are culturally appropriate and targeted at lowering the incidence of MDD in Asian populations, which would ultimately enhance mental health and public health initiatives.

## Data Availability

The data that support the findings of this study are available from the online references.
